# Adhesion Improvement of Solvent-Free Pressure-Sensitive Adhesives by Semi-IPN Using Polyurethanes and Acrylic Polymers

**DOI:** 10.3390/polym14193963

**Published:** 2022-09-22

**Authors:** Kwang Hun Park, Dong Yeob Lee, Sung Ha Yoon, Seong Hun Kim, Min Su Han, Seungju Jeon, Yejin Kim, Yong Kwan Lim, Do-Hoon Hwang, Seo-Hyun Jung, Bogyu Lim

**Affiliations:** 1 Center for Advanced Specialty Chemicals, Korea Research Institute of Chemical Technology (KRICT), Ulsan 44412, Korea; 2 Department of Chemistry, and Chemistry Institute for Functional Materials, Pusan National University, Busan 46241, Korea; 3 Think Top Technology (TTT), R&D Center, Cheonan 31245, Korea

**Keywords:** pressure-sensitive adhesives, IPN, solvent-free, PPG, hard segment, creep, holding time

## Abstract

To improve the peel strength and holding time of polypropylene glycol (PPG)-based pressure-sensitive adhesives (PSAs), a semi-interpenetrating polymer network (semi-IPN) was prepared using acrylic polymers. In addition, to prevent air pollution due to volatile organic compound emissions and avoid the degradation of physical properties due to a residual solvent, the PPG-based semi-IPN PSAs were fabricated by an eco-friendly solvent-free method using an acrylic monomer instead of an organic solvent. PPG-based semi-IPN PSAs with different hard segment contents (2.9–17.2%) were synthesized; their holding time was found to depend on the hard segment contents. The peel strength was improved because of the formation of the semi-IPN structure. Moreover, the high degree of hard domain formation in the semi-IPN PSA, derived from the increase in the hard segment content using a chain extender, resulted in a holding time improvement. We believe that the as-prepared PSAs can be used in various applications that require high creep resistance.

## 1. Introduction

Pressure-sensitive adhesives (PSAs) generally adhere to different surfaces instantaneously under low pressure without driving forces such as water, heat, or light [1,2]. They are used in tapes, protective films, and labels, because no adhesive residue is left on the substrate during detachment [3]. The peel strength, tack, and shear strength, which are basic adhesive properties, are determined by the viscoelastic characteristics of PSAs [4,5]. The peel strength is the force required for the PSA to separate from the substrate and is governed by the viscous properties of the PSA. The tack represents the initial instantaneous bonding force. The shear strength represents the cohesive strength of PSA resistance to creep and represents the elastic properties of PSAs [1,6]. 

Solvent-based PSAs that are currently used require solvents to be completely removed in the manufacturing process, and the presence of residual solvents causes the degradation of the physical properties of the PSAs. In addition, volatile organic compounds released by organic solvents cause environmental problems, such as air pollution. In contrast, solvent-free PSAs do not use organic solvents in the manufacturing process; therefore, they can be produced at a reasonable price without adhesive physical property degradation. In addition, such PSAs have been extensively studied because of their eco-friendly manufacturing method that fundamentally mitigates air pollution [3,7,8,9]. 

Currently, the widely used PSA materials are acrylic, natural and synthetic rubbers, and polyurethane (PU) [10]. Although rubber PSAs generally have excellent adhesion properties, they suffer from low oxidative resistance [11]. Acrylic PSAs are mainly synthesized from random copolymers containing monomers with low (butyl acrylate and 2-ethylhexyl acrylate) and high (methyl methacrylate and isobornyl acrylate) glass transition temperatures (T_g_) [9,12]. Acrylic PSAs require chemical crosslinking because of their low cohesive strength, and they have the advantages of high transparency, water resistance, and controllable adhesive properties [6,13,14]. PU-based PSAs have the advantages of low toxicity and environmental friendliness and possess block copolymers of alternating soft and hard segments [10,15]. The soft segment imparts flexible properties at low temperatures because of a low T_g_, whereas the hard segment provides physical crosslinking sites through hydrogen bonding; therefore, solvent resistance and adhesive properties can be controlled by adjusting the ratio of the hard segments [5,10,16,17,18,19]. However, PU-based PSAs have the disadvantages of low tack and peel strength [10,18,20]. The application of polypropylene glycol (PPG)-based PSAs is limited because of their low peel strength and short holding time (maximums of 4 N/cm and 13 min, respectively) [10,18]. To improve these properties of the PPG-based PU PSAs, several studies have been conducted on mixed polyol-based PU PSAs in which PPGs with various molecular weights are used as a composite polyol or two or more polyols, such as PPG and polytetramethylene ether glycol (PTMEG), are mixed [10,18,19,20,21]. For example, Martinez et al. showed that the peel strength of a PU PSA using a mixed polyol containing PPG 2000 (25 wt%) and PPG 450 (75 wt%) was improved to 8.95 N/cm, and the holding time at 25 °C was recorded over 7000 min without failure [18]. In addition, the use of PPG 2000 and PTMEG 2000 in a polyol-based PU PSA at a 1:1 ratio resulted in the highest peel strength of 15.5 N/cm; its holding time was recorded over 3 days without failure [19].

An interpenetrating polymer network (IPN) is a structure in which one or more cross-linked polymer networks are physically entangled without covalent bonds, and the network cannot be separated without bond cleavage [22,23,24,25,26]. UV-curable acrylic PSAs are studied because of their unique crosslinking properties that are essential to forming the IPN [14,27,28,29]. Kim et al. investigated the adhesive properties in relation to the degree of crosslinking after the formation of an IPN structure with different acrylic polymers [28]. In addition, Kim et al. formed a semi-IPN PSA using silicone urethane methacrylates (SiUMA) and acrylic copolymers and studied its adhesive properties [29]. 

In this study, we prepared a novel solvent-free PSA by forming an IPN structure through an eco-friendly method by using an acrylic polymer and PPG PU in order to improve the low adhesion properties of solvent-type PPG-based PSAs. Two polymerization methods were used to fabricate the PSA: Polyaddition polymerization for PU synthesis and free radical polymerization to form the IPN with acrylic copolymers. PU was polymerized using an acrylic monomer without organic solvents. The ratio of the hard segment was controlled according to the PPG molecular weight and the diisocyanate structure. Moreover, the effect of the hard segment content (HS) on the adhesive properties of the PSA was investigated. It was found that the PPG-based PU PSAs possess high creep resistance even at a high temperature of 120 °C because the degree of the hard domains increased owing to the increase in the hard segment and the use of symmetrical diisocyanate.

## 2. Materials and Methods

### 2.1. Materials

Polypropylene glycol (PPG, M_n_ = 2000, 4000, 6000 Da) was purchased from KPX green chemical (Seosan, Korea), VALIKAT Bi-1610 (a bismuth catalyst) was purchased from Umicore (Brussels, Belgium), and hexamethylene diisocyanate (HDI, 99%), isophorone diisocyanate (IPDI, 98% isomers), bis(4-isocyanatocyclohexyl)-methane (H_12_MDI, 90% isomers), diethylene glycol (DEG, 99%), 4-methoxyphenol (MEHQ, 99%), isobornyl acrylate (IBOA), 2-ethylhexyl acrylate (2-EHA), and acrylic acid (AA) were purchased from Sigma Aldrich (Seoul, Korea). Tris(2-hydroxyethyl) isocyanurate triacrylate as a crosslinking agent was purchased from Sartomer (Exton, PA, USA) and AE 700-100 as an NCO-modifier was purchased from Asahi Kasei (Tokyo, Japan). Omnirad 127D was purchased from IGM Resins (Waalwijk, The Netherlands) and used as a photo-initiator.

### 2.2. Structure of Polyurethane and Acrylic Polymers

**Figure 1:**
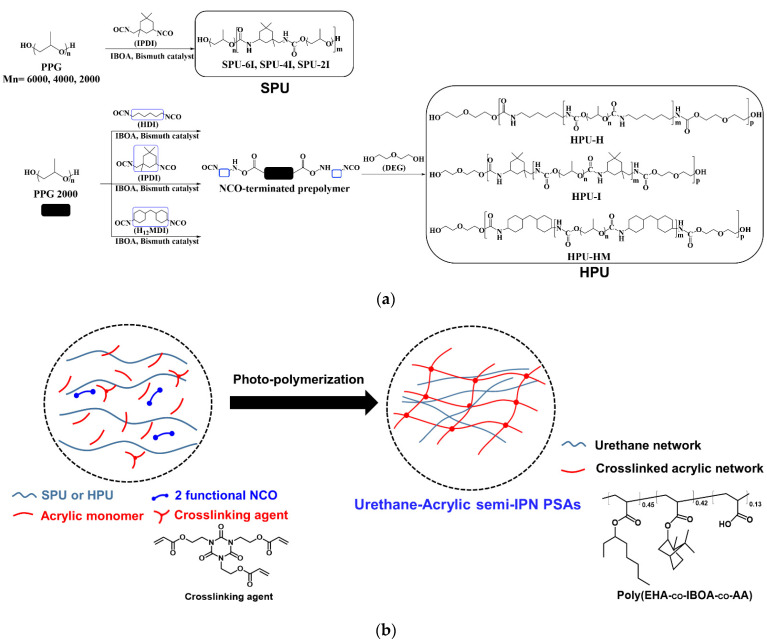
(**a**) Schemes of SPU and HPU, and (**b**) simple schematic of urethane-acrylic semi-interpenetrating polymer network (semi-IPN) PSA synthesis.

The PU structure was designed with OH-terminated urethane prepolymers to react with two NCO functional groups to enhance the entanglement with the acrylic network in the IPN structure. OH-terminated urethane prepolymers were synthesized from PPG, which possesses high compatibility with acrylic polymers, and aliphatic diisocyanates exhibiting high light resistance. By changing the PPG molecular weight, soft PPG urethane prepolymers (SPUs) were synthesized, and diisocyanate was changed in hard PPG urethane prepolymers (HPUs) with DEG as a chain extender. Acrylic polymers were synthesized through free radical polymerization through UV irradiation after mixing with an additional acrylic monomer, a photo-initiator, and a trifunctional crosslinking agent in a urethane prepolymer reaction solution. Figure 1 shows the structures of the SPU, HPU, and IPN.

### 2.3. Synthesis of SPUs

Polymerization was carried out in a 5-neck reactor (MODULAB, Hwaseong, Korea) with a nitrogen inlet, mechanical stirrer, thermocouple, dropping funnel, and reflux condenser. All SPUs were obtained using a one-shot polymerization method. First, excess PPGs (M_n_ = 2000, 4000, and 6000 Da) were poured into the reactor and dried at 90 °C for 1 h under vacuum conditions. Next, calculated amounts of IBOA and MEHQ were added to the reactor as a solvent and an inhibitor, respectively, followed by drying at 80 °C for 2 h under vacuum conditions. Next, the calculated amount of IPDI was added dropwise, and then Bi-1610 (a bismuth catalyst) was added as a catalyst in the reactor, and the reaction was performed at 80 °C in a nitrogen atmosphere. SPUs were obtained when the –NCO group could not be observed via Fourier transform infrared spectroscopy (FT-IR) (Thermo Fisher Scientific Nicolet 6700, Waltham, MA, USA). The target molecular weights of the SPUs were controlled by the molar ratio of OH/NCO. The synthesized SPU samples were denoted as SPU-6I, SPU-4I, and SPU-2I on the basis of the PPG molecular weight, namely, PPG 6000, PPG 4000, and PPG 2000, respectively.

### 2.4. Synthesis of HPUs

Polymerization was carried out in a 5-neck reactor with a nitrogen inlet, mechanical stirrer, thermocouple, dropping funnel and reflux condenser. All the HPUs were obtained using a prepolymer synthesis method. First, PPG 2000 was poured into the reactor and dried at 90 °C for 1 h under vacuum conditions. Next, calculated amounts of IBOA and MEHQ were added to the reactor as a solvent and an inhibitor, respectively, followed by drying at 80 °C for 2 h under vacuum conditions. Next, excess diisocyanates (HDI, IPDI, H_12_MDI) were added dropwise, Bi-1610 (a bismuth catalyst) was added as a catalyst in the reactor, and the reaction was carried out in a nitrogen atmosphere. In the second step, when the –NCO group could not be reduced according to the FT-IR spectral observation, a DEG was added dropwise to the reactor as a chain extender. When the –NCO group was not observed by FT-IR spectroscopy, HPUs were obtained. The synthesized samples were denoted as HPU-H, HPU-I, and HPU-HM according to the molecular structure of the diisocyanates (HDI, IPDI, and H_12_MDI). 

### 2.5. Fabrication of Urethane-Acrylate Semi-IPN PSAs

PSA syrups contained SPU or HPU solution (45%), 2-EHA (25 wt%), IBOA (23 wt%), AA (7 wt%), or tris(2-hydroxyethyl) isocyanurate triacrylate (0.6 phr) as a crosslinking agent, omnirad 127D (2.0 phr) as a photo-initiator, and AE 700-100 as an NCO modifier (0.3 phr). The PSA syrups were coated with a Baker film applicator (OCEAN SCIENCE, Uiwang, Korea) on a PET film. After covering the release film, it was photo-polymerized under total radiation of 2400 mJ. Finally, thermal curing was performed at 60 °C for 3 days to prepare semi-IPN PSAs.

### 2.6. Characterization

^1^H-NMR analysis of the SPUs and HPUs was conducted using a Bruker Avance III HD 300 MHz spectrometer. FT-IR spectra were recorded using a Thermo Fisher Scientific Nicolet 6700 (OMNIC 8.3, Waltham, MA, USA) over the wavenumber range of 4000−550 cm^−1^ with 32 scans and a 4 cm^−1^ resolution. The peak areas for ordered hydrogen-bonded carbonyl groups (A_O_), disordered bonded carbonyl groups (A_D_), and free carbonyl groups (A_F_) of the urethane prepolymer were analyzed for absorption peaks through multiple peak analysis of origin at 1760–1660 cm^−1^. In addition, the relative contents of the ordered hydrogen bonds (R_O_) and disordered hydrogen bonds (R_D_) were calculated using Equations (1) and (2), respectively.

R_O_ = ((A_O_)/(A_O_ + A_D_ + A_F_)) × 100%, (1)

R_D_ = ((A_D_)/(A_O_ + A_D_ + A_F_)) × 100%, (2)

The molecular weight and molecular weight distribution were determined through gel permeation chromatography (GPC) (Agilent Technologies 1260, Santa Clara, CA, USA) with THF (SAMCHUN CHEMICALS, Seoul, Korea) as the mobile phase (1.0 mL min^−1^) at 40 °C and calibrated with PS standards (Agilent, Santa Clara, CA, USA). The hydroxyl value (OHV, OH value) was calculated according to the ASTM E 1899-08 standard. Thermogravimetric analysis (TGA) was performed on a Thermos gravimetric analyzer TA instrument TGA Q500 (WATERS, New Castle, DE, USA). Differential scanning calorimetry (DSC) was performed on a TA instrument DSC Q2000 (WATERS, New Castle, DE, USA) at a heating rate of 10 °C/min in an N_2_ atmosphere. Then, 180° peel strengths were measured using a universal testing machine (Shimadzu Scientific Korea, AGS-X, Kyoto, Japan). Ball tack tests were performed according to the JIS B 1501 standard using TO-730 (Test one). Holding time tests were performed under conditions of 25 °C × 1 kg × 1 h and 120 °C × 1 kg × 1 h.

## 3. Results and Discussion

### 3.1. SPU and HPU Syntheses

To synthesize eco-friendly solvent-free PSAs, SPUs and HPUs, which are urethane prepolymers, were fabricated using acrylic monomer IBOA as a substitute for organic solvents. In addition, IBOA was used in the synthesis of solvent-free semi-IPN PSAs because it not only acts as a solvent but also participates in the formation of the IPN structure. As shown in Table 1, SPUs with similar molecular weights of approximately 46,000 g/mol were synthesized by adjusting the OH/NCO ratio according to the PPG molecular weight (M_n_ = 6000, 4000, and 2000 Da). Results showed no significant difference in the adhesion properties with the variation of the PPG molecular weight (M_n_ = 6000, 4000, and 2000 Da); therefore, PPG 2000 was used in the HPU synthesis, and the change in the adhesive properties with respect to the diisocyanate structure was observed. This will be discussed in the next section. HPUs were synthesized according to the aliphatic diisocyanate structures (HDI, IPDI, and H_12_MDI), by fixing the OH/NCO ratio to 1.01. As an OH group is required at the terminus of the prepolymer to form the IPN structure, it was confirmed that an OH group exists at the terminus of the synthesized urethane prepolymers through OHV calculation. To investigate the effect of the HS on the adhesive properties of the PPG-based PSA, the HS was calculated as the ratio of the weight of diisocyanate and DEG to the weight of PPG, diisocyanate, and DEG [18].

### 3.2. SPU-Based Urethane-Acrylic Semi-IPN PSA

#### 3.2.1. ^1^H-NMR Analysis

**Figure 2:**
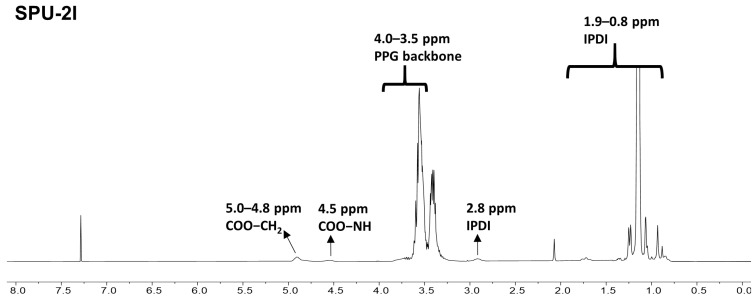
^1^H-NMR spectrum of SPU-2I.

The chemical composition of the SPUs was analyzed by ^1^H-NMR spectroscopy. Figure 2 shows the NMR spectrum of SPU-2I synthesized using PPG 2000. The amide peak of the urethane group (COO–NH) and the proton peaks from PPG bound to the urethane group (COO–CH_2_, COO–CH) were observed at approximately 4.50 ppm and 4.8 to 5.0 ppm, respectively. The backbone proton peaks of PPG were confirmed at approximately 4.0–3.5 ppm, while the proton peaks of IPDI were found at 2.8 and 1.9–0.8 ppm, respectively [30,31]. Figure S1 shows the NMR spectra of SPU-6I and SPU-4I synthesized using PPG 6000 and PPG 4000, respectively. The proton peaks were observed at the same position as those of SPU-2I, and the peaks corresponding to the amide group of the urethane moiety (COO–NH) and the COO–CH_2_, COO–CH proton peaks of PPG were observed.

#### 3.2.2. FT-IR Analysis

**Figure 3:**
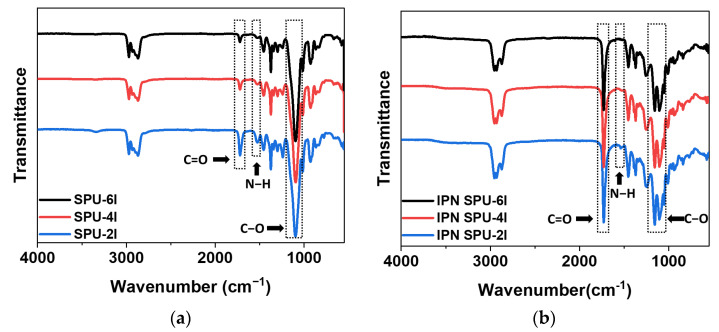
FT-IR spectra of the (**a**) SPU samples and (**b**) SPU-based semi-IPN PSA samples.

For the structural characterization of the SPU-6I, SPU-4I, and SPU-2I, FT-IR spectroscopy was performed (Figure 3). The C=O stretching, N-H bending, and C–O stretching peaks from the urethane group of the SPUs were observed at 1680–1740, 1500–1640, and 1100 cm^−1^, respectively [32,33,34]. As shown in Figure 3a, as the PPG molecular weight increased from 2000 to 6000, the peak intensities of the N-H and C=O groups decreased for the SPU samples. The possible reason for this tendency is the number of urethane repeating groups in the SPU that decreased with an increase in the PPG molecular weight. Therefore, SPU-6I exhibited the lowest C=O stretching and N-H bending peak intensities.

After synthesizing the IPN SPU using the SPU prepolymer, we investigated the formation of the urethane-acrylic semi-IPN through FT-IR analysis. Figure 3b shows the FT-IR spectra of the SPU-based semi-IPN PSAs. The characteristic peaks of the urethane group appeared at positions similar to those for the SPU samples. However, a stronger C=O peak was observed near 1740 cm^−1^, and the C–O peaks of the acrylate group were observed at 1100–1200 cm^−1^. In addition, because the C=C peak of the acrylic monomer was not observed at 1630–1660 cm^−1^, it was concluded that all acrylic monomers reacted.

The curve-fitting results for the C=O stretching peak of the SPU samples are shown in Figure S2a–c. The formed hydrogen bonds are classified into disordered hydrogen bonds and ordered hydrogen bonds; they appeared at 1700 and 1685 cm^−1^, respectively. Furthermore, the free C=O peak, which represents non-hydrogen bonding, appeared at 1720 cm^−1^ [35,36,37,38]. The relative content of the hydrogen bonds in the SPU samples was calculated using Equations (1) and (2) [37]. As shown in Table 2, as the PPG molecular weight increased, the N-H peak area decreased because HS was lowered. Therefore, SPU-6I possessed the lowest hydrogen bond content (32.4%), whereas SPU-2I had the highest hydrogen bond content (44.0%). In addition, the disordered hydrogen bond content was higher than the ordered hydrogen bond content, which was expected because of the asymmetric structure of IPDI and steric hindrance caused by the methyl side chain [37,38,39].

#### 3.2.3. Thermal Analysis

**Figure 4:**
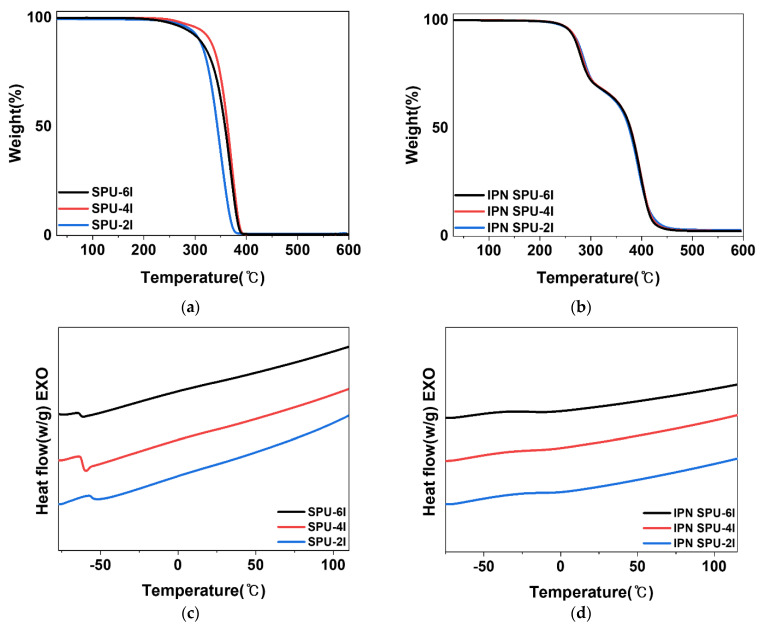
(**a**) TGA curves of the SPUs and (**b**) SPU-based semi-IPN PSAs. (**c**) DSC curves of the SPUs and (**d**) SPU-based semi-IPN PSAs.

The thermal properties of the SPUs and IPN SPUs were characterized by TGA and DSC (Figure 4 and Table 3). Because the SPUs were synthesized by the reaction between PPG and IPDI without a chain extender, the hard segment in the SPU chain is expected to be short. Therefore, because the formation of a hard domain was difficult and micro-phase separation rarely occurred, only thermal decomposition by PPG, a soft segment (T_S_), was observed at 349–371 °C (Figure 4a) [20,40]. Figure 4b shows the TGA curves of the SPU-based semi-IPN PSAs. The thermal decomposition temperature of the urethane network (T_U_) and the thermal decomposition temperature of the acrylic polymer network (T_A_) were approximately 280 and 400 °C, respectively. In addition, the IPN was expected to be formed by crosslinking because the residue remained even at a high temperature of 600 °C [24].

DSC was performed to investigate the relationship between T_g_ and the HS and structure of the hard segments. In Figure 4c, the T_g_ of the SPU samples increased in the order of SPU-2I (−58.4 °C) > SPU-4I (−61.5 °C) > SPU-6I (−61.7 °C) with an increase in the HS [41]. This is due to the restriction of the PPG backbone movement because the amorphous hard segments are distributed in the soft matrix with an increase in the HS.

The DSC curves of the semi-IPN PSA with the SPU are shown in Figure 4d. The T_g_ of the SPU-based IPN PSA samples was significantly increased by approximately 40 °C or more in relation to that of the SPU under the influence of physical entanglement, which is an inherent phenomenon induced by the IPN structure. This indicates that the IPN structures with urethane and acrylic networks were successfully formed [22]. In addition, the IPN SPU-2I sample prepared from SPU-2I with the highest HS exhibited the highest T_g_ of −12 °C.

#### 3.2.4. Adhesive Properties of SPU-Based Semi-IPN PSAs

The adhesion performance of the SPU-based semi-IPN PSAs was evaluated by conducting the ball tack, 180° peel strength, and holding time tests at 25 and 120 °C (Table 4). The tack is the ability to adhere within a short time upon the application of a low contact pressure without using energy, such as that derived from solvent addition or heating. In the ball tack test, a large ball number indicates high tack ability. In the ball tack test result of the SPU-based semi-IPN PSAs, all three samples showed the same value; therefore, a relationship between the HS or PPG molecular weight and tack was not observed. This was expected because the three samples had similar acrylic networks in the IPN structure. 

Similarly, the peel strength test results also indicated similar values for all three samples; therefore, the effect of the PPG molecular weight or HS on peel strength was not observed; however, all samples had peel strength values of >2000 g/inch. Therefore, it was confirmed that the PPG-based PSAs exhibit improved peel strength because of the formation of the IPN structure.

In contrast, the holding time is a value that indicates the cohesive strength of PSAs and is affected by the HS [42]. As shown in Table 4, in the SPU-based PSA samples, creep occurred within 8 min at 120 °C because hard domains were rarely formed owing to the low HS. Therefore, the IPN SPU-6I sample with the lowest HS exhibited the shortest holding time of 6 min. To improve the holding time of PSAs, the HPU-based semi-IPN PSAs with increased HS were prepared by introducing DEG as a chain extender and various diisocyanates such as HDI, IPDI, and H_12_MDI [43].

### 3.3. HPU-Based Urethane-Acrylic Semi-IPN PSA

#### 3.3.1. ^1^H-NMR Analysis

**Figure 5:**
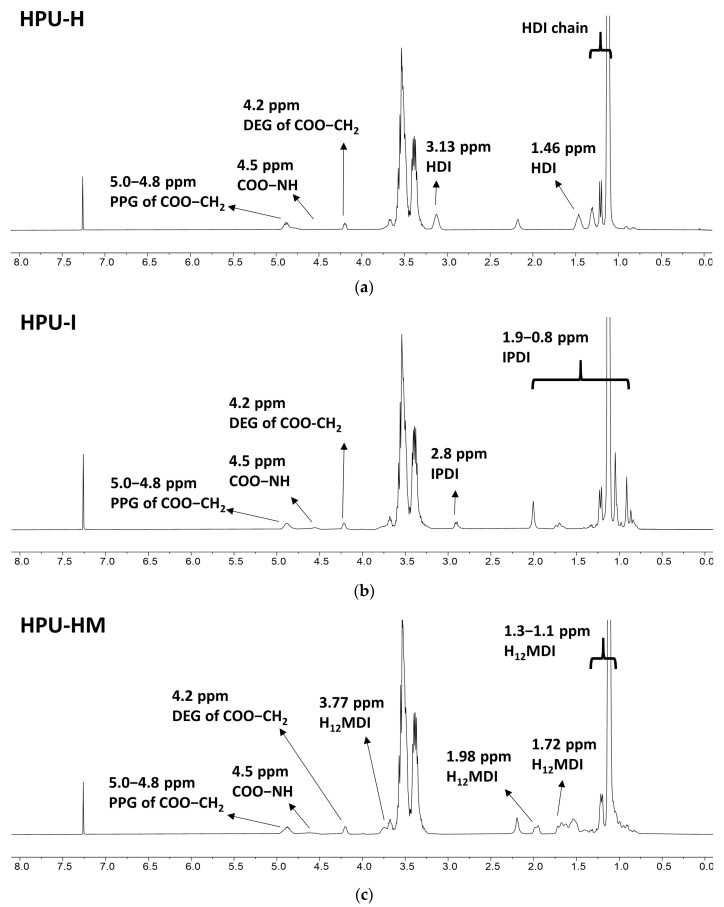
^1^H-NMR spectra of (**a**) HPU-H (**b**) HPU-I and (**c**) HPU-HM.

The chemical compositions of HPU-H, HPU-I, and HPU-HM prepared with three diisocyanates (HDI, IPDI, and H_12_MDI) were analyzed through ^1^H-NMR spectroscopy. In all the HPU NMR spectra, the proton peaks next to urethane generated from PPG and DEG were identified at 4.8–5.0 ppm and 4.2 ppm, respectively [30,44]. Figure 5a shows the ^1^H-NMR spectrum of HPU-H. The proton peaks corresponding to HDI were observed at 3.13 and 1.46 ppm [30,45]. In Figure 5c, the proton peaks assigned to H_12_MDI and the cyclic and symmetric structures appeared at 3.77, 1.98, 1.72, and 1.30–1.10 ppm [46].

#### 3.3.2. FT-IR Analysis

**Figure 6:**
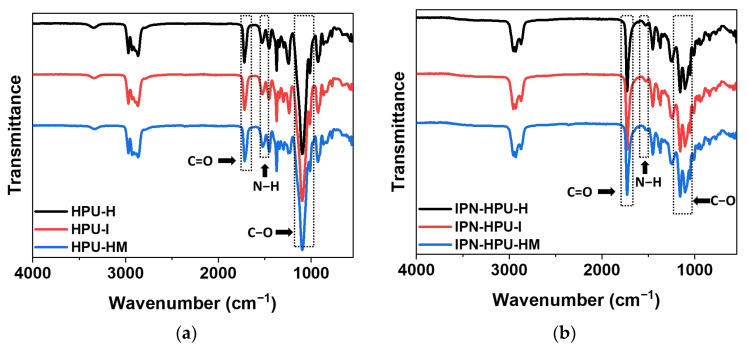
FT-IR spectra of the (**a**) HPU samples and (**b**) HPU-based semi-IPN PSAs.

For the structural characterization of the HPUs prepared with three different diisocyanates, FT-IR spectroscopy was performed (Figure 6). Figure 6a shows the FT-IR spectra of the HPUs before IPN structure formation; C=O stretching, N-H bending, and C–O stretching peaks of the urethane group were observed at the same position as those of the SPUs. However, the intensity of the peaks attributed to the urethane groups (C=O and N-H) in HPUs was higher than that for SPU-2I [32,33,34].

Figure 6b shows the FT-IR spectra of the HPU-based semi-IPN PSAs. The C=O and C–O peaks of the urethane group were located at positions similar to those of the SPU-based IPN PSA samples. However, the intensity of the N-H peak slightly increased in relation to that of the SPU-based IPN PSAs owing to the increase in the number of repeating units of the urethane group upon the use of DEG.

The curve fitting results for the C=O stretching peak of the HPU samples are shown in Figure S2d–f. The effect of the three diisocyanates on hydrogen bonding was investigated by calculating the relative hydrogen bond content of the carbonyl groups of the HPU samples (Table 5). Unlike the SPU samples, for which the bond content was determined based on HS, for the HPU samples, the free carbonyl groups were determined according to the diisocyanate structure. HPU-H with HDI diisocyanate showed the highest ordered hydrogen bond content and a total hydrogen bond content of 37%. In contrast, HPU-I with IPDI showed the lowest hydrogen bond content. This can be explained by the fact that HDI has a packed arrangement between hard segments because of its linear and symmetrical structure, but IPDI has an asymmetric structure and possesses methyl substituents that cause steric hindrance; therefore, packing between the hard segments is unsatisfactory [37,39]. Thus, HPU-HM with H_12_MDI having a bulky semi-symmetrical structure showed an intermediate value between those for HPU-H and HPU-I.

#### 3.3.3. Thermal Analysis

**Figure 7:**
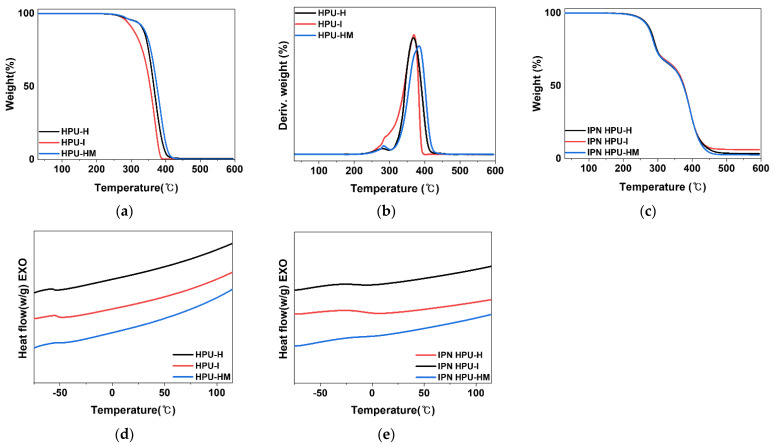
(**a**) TGA curves of the HPUs; (**b**) DTG curves of the HPUs; (**c**) HPU-based semi-IPN PSAs; (**d**) DSC curves of the HPUs; (**e**) DSC curves of the HPU-based semi-IPN PSAs.

The thermal properties of the HPUs and IPN HPUs were characterized through TGA, derivative thermogravimetric analysis (DTG), and DSC. Figure 7a shows the TGA curves of the HPU samples. Because the HPU samples had hard segments of critical length owing to the introduction of DEG, a chain extender, two degradation temperatures due to micro-phase separation were observed [18,20,40]. As shown in Table 6, the thermal decomposition of the hard segment (T_H_) in the DTG curve of the HPU samples appeared at 280 °C, and then the thermal decomposition of the soft segment occurred slowly [18,40]. In HPU-H and HPU-HM, the hard and soft segment peaks were well separated; however, in the case of HPU-I, peak separation did not occur clearly, and a shoulder peak was observed (Figure 7b). This is due to the fact that almost no micro-phase separation occurs because HPU-I has an asymmetrical IPDI structure. Therefore, it was found that the symmetry of the diisocyanate structure affected micro-phase separation.

Figure 7c shows the TGA curves of the HPU-based semi-IPN PSAs; it is expected that the IPN was formed by crosslinking. In addition, T_U_ and T_A_ were observed at approximately 290 and 390 °C, respectively.

According to the DSC results of the HPU samples, which were similar to those of the SPU samples, T_g_ increased with an increase in the HS (Figure 7d). In particular, by comparing the DSC results for SPU-2I and HPU-I having the same PPG molecular weight and same diisocyanate (IPDI), the T_g_ of HPU-I (−52.7 °C) was higher than that of SPU-2I. On the other hand, among the HPU samples, HPU-H with the lowest HS showed the lowest T_g_ of −55.8 °C. Therefore, the HS affected the T_g_ in the PPG-based PSAs.

The DSC curves after the formation of the urethane-acrylic IPN structure, using the HPU prepolymer, are shown in Figure 7e. The HPU-based semi-IPN PSA showed a higher T_g_ than the HPU prepolymer; therefore, it could be expected that the IPN structure was well formed. In particular, the T_g_ of IPN HPU-HM was the highest at −5 °C, which is attributed to H_12_MDI with a non-planar structure and high aspect ratio. Because the H_12_MDI diisocyanate does not form planar hydrogen bonds, HPU-HM showed the highest T_g_ because the degree of dispersion in the soft matrix is high during the formation of randomly arranged short-length hard domains.

#### 3.3.4. Adhesive Properties of HPU-Based Semi-IPN PSAs

The adhesion performance of the HPU-based semi-IPN PSA was evaluated by conducting the ball tack, 180° peel strength, and holding time tests at 25 and 120 °C (Table 7). The ball tack test result of the HPU-based semi-IPN PSA showed #4, which is the same value as that observed for the SPU-based semi-IPN PSA. This was expected because they had similar acrylic networks in the IPN structure.

As shown in Table 7, the peel strength was 1750–2030 g/inch, which was also similar to that of the SPU-based semi-IPN PSA. However, the holding time at 120 °C was significantly increased. Although IPN HPU-I had a high HS, the hard domain was not well-formed, resulting in a holding time of only 11 min; however, the holding time of the IPN HPU-H was 51 min, which was significantly higher than that of the SPU-based semi-IPN PSA. In particular, for IPN HPU-HM with the highest HS, no creep occurred during the holding time test, indicating excellent creep resistance and cohesion even at a high temperature of 120 °C.

## 4. Conclusions

To systematically investigate the effect of the HS on the adhesion properties of PSAs, the HS of the SPU and HPU samples was controlled by changing the PPG molecular weight (M_n_ = 6000, 4000, and 2000 Da) and the diisocyanate structure (HDI, IPDI, and H_12_MDI). In addition, solvent-free urethane-acrylate semi-IPN PSAs were prepared through photo-polymerization to improve the adhesion properties of the PU-based PSA. 

The analysis of the hydrogen bond structure of the carbonyl group through FT-IR spectroscopy revealed that the SPU samples showed a high relative hydrogen bond content as the HS increased with a decrease in the PPG molecular weight. However, the relative hydrogen bond content of the HPU samples was more dependent on the diisocyanate structure than HS. The HPU sample to which IPDI was introduced showed an ordered hydrogen bond content of only 8% owing to the asymmetric structure and steric hindrance of IPDI. In contrast, in the HPU samples containing a symmetric diisocyanate, HDI, or H_12_MDI, the ordered hydrogen bond contents were 16.3% and 14.5%, respectively, suggesting that the hard domain was well formed. Therefore, the adhesion properties could be controlled via controlling the hard domain formation by tuning the diisocyanate structure used in the synthesis of the PU-based PSAs.

Thermal analysis results indicated that a chain extender is essential for the formation of a hard domain, and the packing of the hard segment and the degree of micro-phase separation were determined according to the diisocyanate structure. In the SPU-based PSA, T_g_ tends to increase with a decrease in the PPG molecular weight because the movement of the PPG backbone is restricted by the increase in the HS. Furthermore, in the case of the HPU-based PSAs with long hard segments, T_g_ increased with an increase in the HS because of the introduction of the chain extender. Thus, the HS was found to directly affect the T_g_, which is related to cohesive strength.

The adhesive property analysis result suggested that the peel strength of the SPU or HPU-based urethane-acrylate semi-IPN PSAs was improved to approximately 2000 g/inch, as expected. In addition, the holding time of the HPU-based semi-IPN PSAs at 120 °C was longer than that of the SPU-based semi-IPN PSAs owing to the high cohesion. In particular, IPN HPU-HM with the highest HS showed impressive non-creep results in the holding time test at 120 °C.

Consequently, the low peel strength, which is the main disadvantage of the PU-based PSAs, was improved through the formation of the urethane-acrylate semi-IPN, and the adhesion properties were adjusted by controlling the degree of hard domain formation by tuning the diisocyanate structure. Moreover, because no organic solvent was used during the synthesis, an environmentally friendly PU-based PSA was realized. The as-prepared, novel, solvent-free PU PSA can be used in applications such as medical labels and tapes that can maintain adhesion over a wide temperature range.

## Figures and Tables

**Table 1:** Synthesis conditions, molecular weights, OHVs, and HS of the prepolymer samples.

Urethane Prepolymer	Polyol	Diisocyanate	Molar Ratio (PPG/Diisocyanate/DEG)	Mw (g/mol)	PDI	OHV (KOH mg/g)	Hard Segment Content (HS)
SPU-6I	PPG 6000	IPDI	1.30:1:0	46,100	1.73	2.20	2.9%
SPU-4I	PPG 4000		1.10:1:0	49,400	1.89	1.73	5.1%
SPU-2I	PPG 2000		1.02:1:0	46,100	1.95	1.55	11.0%
HPU-H	PPG 2000	HDI	0.71:1:0.3	137,000	1.71	0.73	12.4%
HPU-I		IPDI	0.71:1:0.3	105,000	1.93	1.43	15.2%
HPU-HM		H_12_MDI	0.71:1:0.3	111,000	1.86	1.15	17.2%

**Table 2:** Relative contents of hydrogen bonding of carbonyl groups in SPU samples.

Sample	N-H Peak Area	Relative Absorbance of C=O Groups in Polyurethane		
		Free C=O (A_F_, 1720 cm^−1^)	Disordered H-Bond C=O (A_D_, 1700 cm^−1^)	Ordered H-Bond C=O (A_O_, 1685 cm^−1^)
SPU-6I	119.5	67.6%	32.4%	-
SPU-4I	151.6	64.6%	35.4%	-
SPU-2I	347.5	56.0%	32.0%	12.0%

**Table 3:** Thermal properties of the SPUs and semi-IPN PSAs with SPUs.

	T_H_ (°C)	T_S_ (°C)	T_g_ (°C)	T_U_ (°C)	T_A_ (°C)	Urethane (%)	Acrylate (%)
SPU-6I	-	369.6	−61.7	-	-	-	-
SPU-4I	-	371	−61.5	-	-	-	-
SPU-2I	-	349	−58.4	-	-	-	-
IPN SPU-6I	-	-	−19.7	279.9	397.8	32.1	64.9
IPN SPU-4I	-	-	−17.5	282.4	397.7	32.6	64.1
IPN SPU-2I	-	-	−12	285.8	394.3	32.1	64.4

**Table 4:** Adhesion performance of the SPU-based semi-IPN PSAs.

Sample	Ball Tack	Peel Strength (g/inch)	Holding Time (25 °C × 1 kg × 1 h)	Holding Time (120 °C × 1 kg × 1 h)
IPN SPU-6I	#4	2190	Non-creep	6 min
IPN SPU-4I	#4	2070	Non-creep	8 min
IPN SPU-2I	#4	2120	Non-creep	8 min

**Table 5:** Relative contents of hydrogen bonding of carbonyl group in the HPU samples.

Sample	N-H Peak Area	Relative Absorbance of C=O Groups in Polyurethane		
		Free C=O (A_F_, 1720 cm^−1^)	Disordered H-Bond C=O (A_D_, 1700 cm^−1^)	Ordered H-Bond C=O (A_O_, 1685 cm^−1^)
HPU-H	426.6	63.0%	20.7%	16.3%
HPU-I	443.5	71.1%	21.9%	8.0%
HPU-HM	486.3	64.1%	21.4%	14.5%

**Table 6:** Thermal properties of the HPUs and HPU-based semi-IPN PSAs.

Sample	T_H_ (°C)	T_S_ (°C)	T_g_ (°C)	T_U_ (°C)	T_A_ (°C)	Urethane (%)	Acrylate (%)
HPU-H	281.6	368.0	−55.8	-	-	-	-
HPU-I	286.0	369.0	−52.7	-	-	-	-
HPU-HM	283.0	385.0	−50.3	-	-	-	-
IPN HPU-H	-	-	−16.0	290.1	393.9	33.1	62.7
IPN HPU-I	-	-	−7.8	286.5	392.5	32.0	61.0
IPN HPU-HM	-	-	−5.0	286.0	393.9	32.6	64.0

**Table 7:** Adhesive properties of HPU-based semi-IPN PSAs.

Sample	Ball Tack	Peel Strength (g/inch)	Holding Time (25 °C × 1 kg × 1 h)	Holding Time (120 °C × 1 kg × 1 h)
IPN HPU-H	#4	2030	Non-creep	51 min
IPN HPU-I	#4	1990	Non-creep	11 min
IPN HPU-HM	#4	1750	Non-creep	Non-creep

## Data Availability

Not applicable.

## Supplementary Materials

The following supporting information can be downloaded at: https://www.mdpi.com/article/10.3390/polym14193963/s1, Figure S1: 1H-NMR spectra of (a) SPU-6 and (b) SPU-4I; Figure S2: Curve fitting results for the C=O stretching peak of (a) SPU-6I, (b) SPU-4I, (c) SPU-2I, (d) HPU-H, (e) HPU-I, and (f) HPU-HM.
