# Supplementary figures and images for: Adhesion Improvement of Solvent-Free Pressure-Sensitive Adhesives by Semi-IPN Using Polyurethanes and Acrylic Polymers
Source: Polymers (Basel). 2022 Sep 22;14(19):3963. doi: 10.3390/polym14193963 (PMC9571291; doi:10.3390/polym14193963)

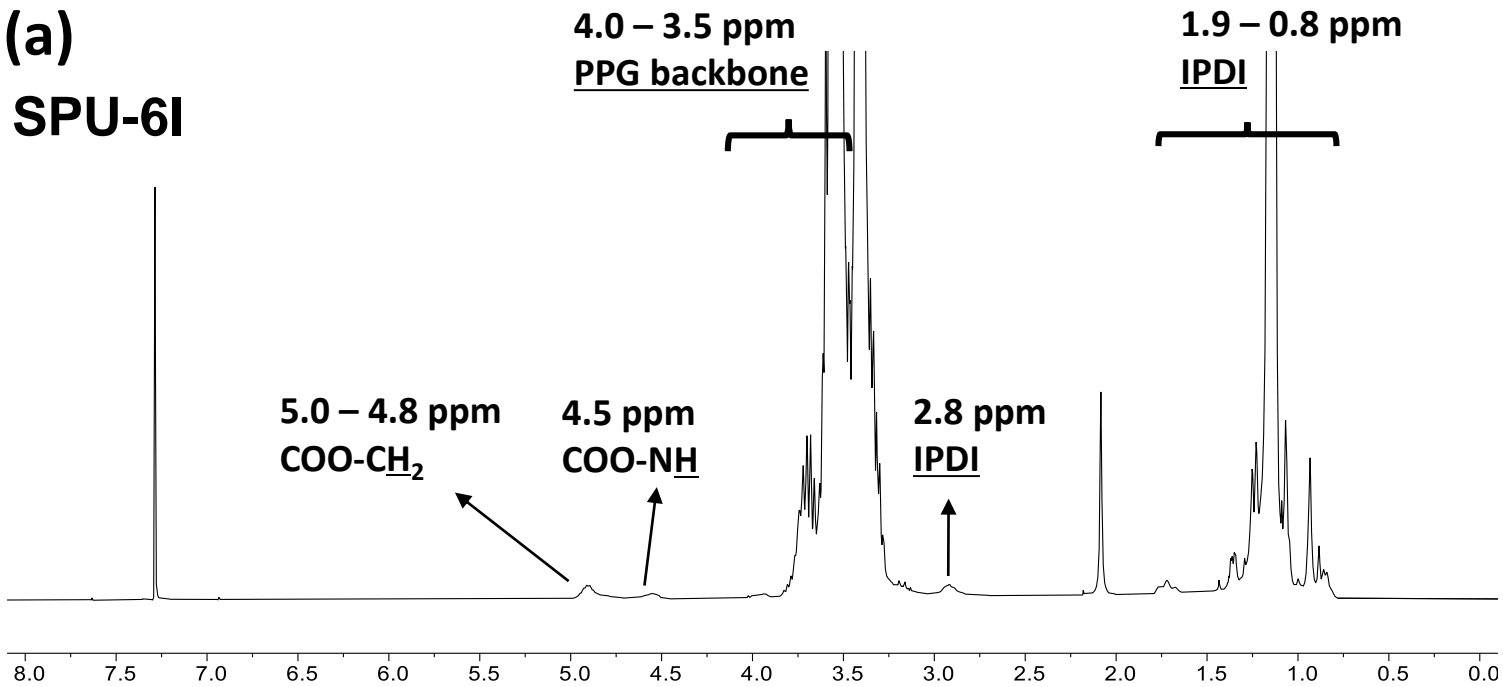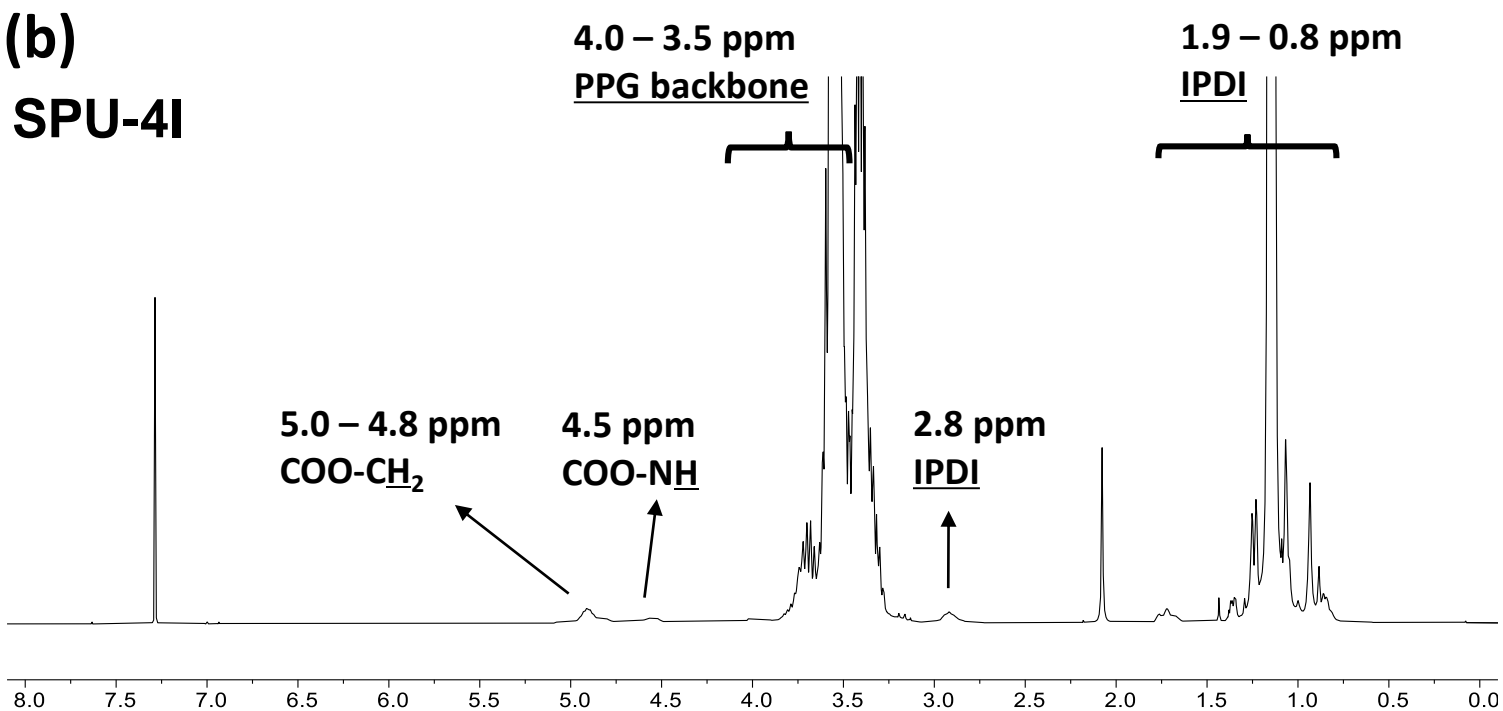

Supplement: Supplementary file 1 [file polymers-14-03963-s001.zip › Figure S1.pdf]

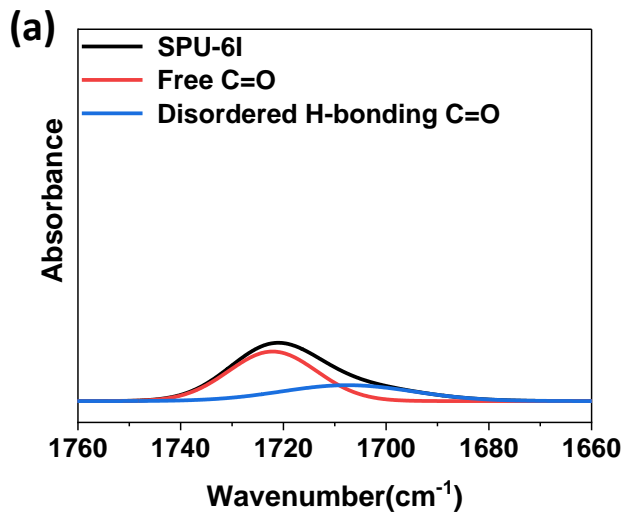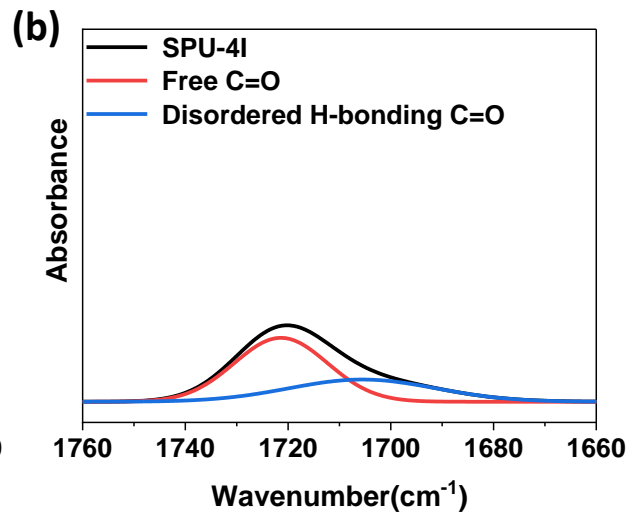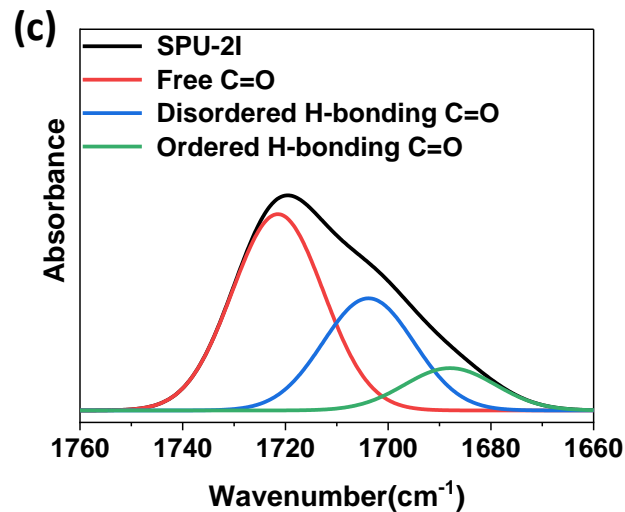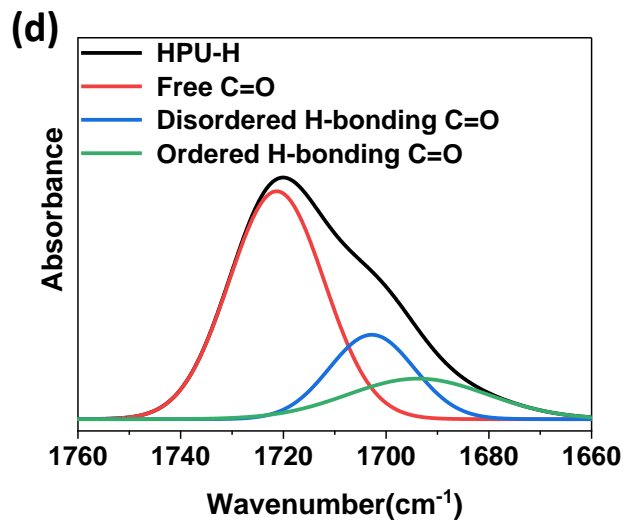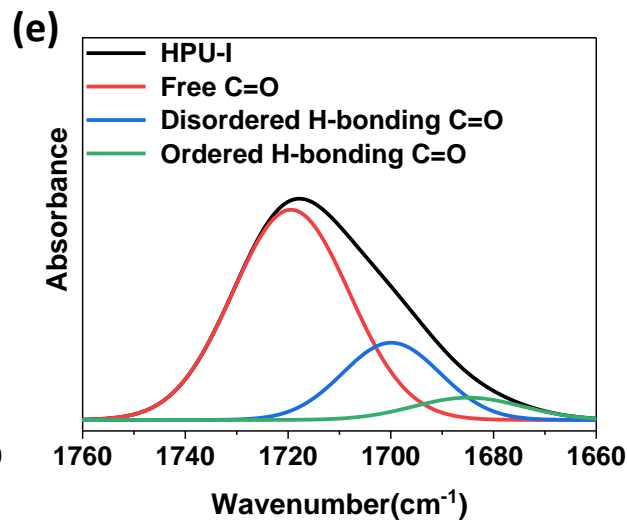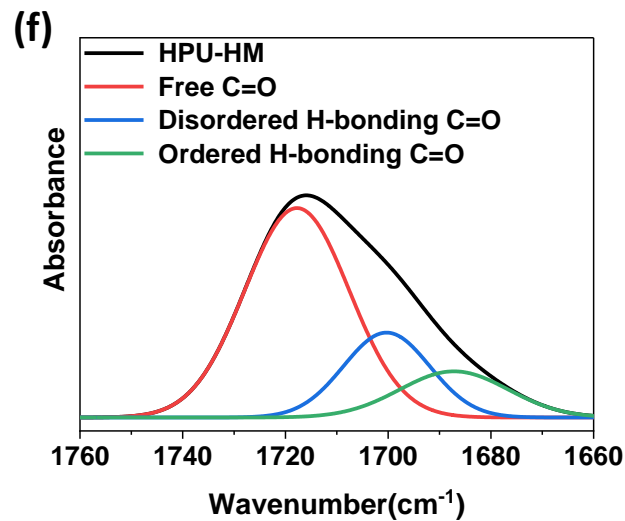

Supplement: Supplementary file 1 [file polymers-14-03963-s001.zip › Figure S2.pdf]
